# Ionotropic receptors signal host recognition in the salmon louse (*Lepeophtheirus salmonis*, Copepoda)

**DOI:** 10.1371/journal.pone.0178812

**Published:** 2017-06-05

**Authors:** Anna Z. Komisarczuk, Sindre Grotmol, Frank Nilsen

**Affiliations:** Sea Lice Research Centre, Department of Biology, University of Bergen, Bergen, Norway; USDA-ARS Beltsville Agricultural Research Center, UNITED STATES

## Abstract

A remarkable feature of many parasites is a high degree of host specificity but the mechanisms behind are poorly understood. A major challenge for parasites is to identify and infect a suitable host. Many species show a high degree of host specificity, being able to survive only on one or a few related host species. To facilitate transmission, parasite’s behavior and reproduction has been fine tuned to maximize the likelihood of infection of a suitable host. For some species chemical cues that trigger or attract the parasite in question have been identified but how metazoan parasites themselves receive these signals remains unknown. In the present study we show that ionotropic receptors (IRs) in the salmon louse are likely responsible for identification of a specific host. By using RNAi to knock down the expression level of different co-receptors, a significant change of infectivity and settlement of lice larvae was achieved on Atlantic salmon. More remarkably, knock down of the IRs changed the host specificity of the salmon louse and lice larvae settled at a significant rate on host that the wild type lice rejected within minutes. To our knowledge, this has never before been demonstrated for any metazoan parasite. Our results show that the parasites are able to identify the host quickly upon settlement, settle and initiate the parasitic life style if they are on the right host. This novel discovery opens up for utilizing the host recognition system for future parasite control.

## Introduction

Chemical sensing plays a crucial role for organisms in order to transmit signals from the environment to an individual resulting in changed behavior or physiological responses. Peripheral detection and recognition of chemical cues depends on membrane receptor proteins that bind specific ligands in the environment and convert this interaction into cellular processes. In invertebrates, the vast majority of environmental stimuli is recognized by members of two evolutionarily related chemosensory receptor families, the odorant receptors (ORs) and the gustatory receptors (GRs) [1–4]. Recently Benton et al (2009) [5] identified a novel type of chemosensory receptors in *Drosophila* that where given the name Ionotropic Receptors (IRs). IRs are a group of evolutionary conserved Ionotropic Glutamate Receptors (iGluRs) and increase the diversity of the chemosensory system. IRs have later been found in olfactory organs across Proteostoma [5–15], indicating that IRs are likely the most ancient chemosensory receptors [16]. The IRs share characteristic features with iGluRs but their ligand-binding domains are considerably more variable and lack one or more of the conserved residues resulting in an inability to bind glutamate. This high variation in the LBD domain and protein localization to ciliated endings of the sensory neurons indicates that they can recognize distinct molecules present in external environment [5].

Parasites show a varying degree of host specificity where some are specialist to live on one or a few related host species, whereas others are generalists and live on a wide diversity of host species. A high degree of host specificity can be explained by parasite mortality after settlement on wrong hosts (e.g. by some sort of immunity) or that a parasite is able to distinguish between hosts and then settle on suitable hosts only. By using chemical cues a parasite could assess a potential host quickly and make a decision whether to stay or go. Previous studies have shown that parasites are able to recognize host derived odors and make a decision about infection [17,18]. However, it is unknown which receptors mediate this signal in any parasite.

The salmon louse (*Lepeophtheirus salmonis*) is a marine ectoparasite on salmonids and Atlantic salmon (*Salmo salar*) is one of the main host in the Atlantic [19]. The life cycle consist of eight instars [20] where three are free-living lecitotrophic larva (i.e. nauplia I, nauplia II and the infectious copepodid) and the remaining five are parasitic on salmonids. Salmon louse and similar parasites feed on the mucus, skin and blood of the host [21] and represents large economical loss for the salmon farming industry and a significant threat to wild salmonids [22]. Lice control has largely relied on anti-sea lice medicine resulting in an emerging resistance development [23]. Hence, new tools to combat sea lice are needed.

Specialized ectoparasites such as *L*. *salmonis* have optimized the behavior to be present where there is a high chance for encountering a host. The infectious copepodids are positive phototactic and they will stay at the surface area where the salmonid hosts prefer to be.

Host location by the copepodids involves mechanical, visual and chemical cues, which operate together in order to ensure correct host recognition, landing and settlement [24,25]. Previous studies have shown that long-range location and landing process is almost entirely dominated by physical forces induced by factors such as movement of water as a fish swims, light intensity and salinity [25,26]. However, laboratory experiments have demonstrated that chemical cues related to host odor serve to activate copepodids and change their swimming behavior. Bailey *et al*. [27] suggested that this behavior maximize the chance of encounter with a potential host by retaining copepodids in the vicinity of the odor. After landing on the fish, lice use short-range sensory cues engaging both olfaction and taste. Lice directly start to feed on the fish as well as fan with first antenna to ascertain host identity before settlement (own observations).

Olfaction is a critical component of host-seeking behavior. It has been shown that both adults and copepodids are able to detect and respond to chemical stimuli emitted from the skin, flesh and mucus of candidate host species [28–30]. Previous studies have demonstrated that *L*. *salmonis* adults and copepodids displayed high activation and are attracted to odor from Atlantic salmon [27,29]. Using host and non-host fish conditioned water and their extracts, candidate molecules that could serve as attractants and repellents for *L*. *salmonis* have been identified [27,29,30]. However, the mechanisms leading to detection of such cues by chemosensory receptors of salmon louse and the cellular processes that transform signals from peripheral sensory system into behavior patterns resulting in successful host recognition are unknown.

In the present study we attempted to identify *L*. *salmonis* chemoreceptors that could be involved in mediating the host specificity. Exhausted analysis of the salmon louse genome did not reveal any genes belonging to the OR or GR families [31] but a significant number of Ionotropic Glutamate Receptors (iGluRs), including Ionotropic Receptors (IRs), was identified [31]. The lack of a typical insects chemosensory receptors repertoire suggests that IRs can be the main molecules for odor/taste recognition in salmon louse [31]. To approach the hypothesis that IRs are key players in signaling host specificity we combine gene silencing using RNA interference (RNAi) with a behavioral approach. First we demonstrate that a large proportion of the salmon lice IRs are expressed predominantly in the first antenna (i.e. the main olfactory organ) and that these *Ls*IRs are classified by us as antennal receptors [31]. We further assessed their expression levels during the salmon louse life cycle and show that they are most abundant in the infectious copepodids. Moreover, RNAi gene silencing targeted to co-receptors alter the ability of copepodids to distinguish between host/non-host fish, hence, demonstrating a crucial role for the IRs in host recognition signaling.

## Materials and methods

### Ethics statement

The experiments were carried out on salmon louse *Lepeophtheirus salmonis* whereas Atlantic salmon (*Salmo salar*), lumpfish (*Cyclopterus lumpus*) and Ballan wrasse (*Labrus bergylta*) were used only as a host for the experimental animals.

All procedures involving animals were performed according to The Norwegian Animal Welfare legislation. All experiments were approved by The Animal Ethics Committee by The Norwegian Food Safety Authority—Atlantic salmon (approval number 4538), lumpfish (approval number 6207) and Ballan wrasse (approval number 7184).

### Identification and nomenclature of ionotropi receptors (IRs)

IR genes were identified as previously described [31] and named according to a unified nomenclature system for none *Drosophila* IRs as proposed by Croset *et al*. [16]. The names for novel salmon louse specific genes were given numbers from IR320 and upwards (i.e. the next number after last published IR, *L*. *humile Lhum*IR319 [32]).

### Salmon lice culture

A laboratory strain of salmon louse (*Lepeophtheirus salmonis*) was used in all experiments [33] and was maintained on farmed Atlantic salmon (*Salmo salar*) in sea water (34.5 ‰) at 10°C. Before infection, salmon louse were hatched and cultivated to copepodid stage in single well, flow-through incubators, with water supply from the same source [33]. Prior to lice sampling, the salmon were anaesthetized in a mixture of methomidate (5mg/l) and benzocaine (60mg/l) and lice removed with forceps [33]. All experimental procedures were performed according to The Norwegian Animal Welfare legislation.

### dsRNA production for RNAi treatment

To generate dsRNA for each gene, two pairs of primers (without and with T7 promoter extension: TAATACGACTCACTATAGGGAGA) were used to generate PCR product template (S2 Table). Equal amounts of sense and antisense template strands were mixed before dsRNA synthesis. dsRNA synthesis was performed with T7 RNA Polymerase from MEGAscript^®^ RNAiKit (Ambion Inc.) according to manufacturer instructions. Final concentration of dsRNA was measured with spectrometry (NanoDrop Technologies Inc.) and adjusted to 1.5 μg/μl.

### Knock-down of IR co-receptors using RNAi treatment

RNAi was performed according to [34] on nauplii I. Three to five parallel treatments were set up for each gene in all experiments. For fish infection, each experimental group consisted of at least 200 copepodids. For knock-down efficiency, each group consisted of at least 100 animals. For RNAi experiments, animals were incubated in sea water with dsRNA added to final concentration of 15ng/μl. For all experiments control group was accompanied, where animals were treated with dsRNA complementary to CYP185 (850bp) from Atlantic cod (*Gadus morhua*) [35]. Incubations were performed overnight (around 20 hours of soaking). Thereafter, all animals were kept in flow-through incubators, for 7 days—knock-down experiments, 7—9 days for fish infection. Each experiment was repeated three to five times. After each experiment copepodids were fixed in RNAlater (Qiagen) and kept in -20°C until gene expression analysis.

### RNA extraction and cDNA synthesis

Total RNA was extracted from samples previously fixed in RNAlater (Qiagen). Samples were transferred to 1 ml of Tri Reagent (Sigma Aldrich) and homogenized in TissueLyser LT (Qiagen) (2 times 5 minutes at 50Hz). 1.4 mm zirconium oxide beads (Precellys 14) were used for homogenization of naupla I, nauplia II, copepodid, chalimus I and chalimus II stages, and 3 mm stainless steel beads for preadult I, preadult II and adult of both sexes. In case of stages from nauplia I to chalimus II, Tri Reagent was added in two steps to each sample, first 200 μl to cover beads, and before second homogenization remaining 800 μl. The following steps were according to Tri Reagent manufacturer instructions. Total RNA was diluted in DEPC-treated water (Invitrogen) and the final concentration was evaluated with spectrometry (NanoDrop Technologies Inc.). DNase treatment (TURBO, Ambion) and cDNA synthesis (AffinityScript qPCR cDNA Synthesis Kit, Stratagen) were performed directly. For DNase treatment each sample contained 2 μg of total RNA, according to NanoDrop readings. cDNA was diluted four times before storage at -20°C.

### Quantitative RT-PCR

qPCR was performed and analyzed as described before, using as a reference gene the salmon louse elongation factor 1α (*Lsal*EF1α) [36]. Primers used are listed in S3 Table. Each sample was carried out in duplicates. The assay was performed simultaneously for *Lsal*EF1α and the test genes using the same cDNA and master mix (SYBR^®^ Green, Applied Biosystems). Thermal cycling and quantification was done on the Applied Biosystems 7500 Fast Real-Time PCR System in 10 μl reactions in standard conditions (initiation: 50°C 2 min, holding: 95°C 10 min, 40 cycles of 95°C 15 seconds/60°C 1 minute) followed by melting curve evaluation. For relative quantification analysis differences in threshold cycle (ΔC_T_) between gene of interest and *Lsal*EF1α were calculated in relation to the dsCYP185 treated controls. Fold difference of gene expression between control and dsRNA treated groups ware evaluated using Livak method. Independent-Samples T-Test was used to determine if the control and the test groups were differently expressed. A *p*-value of 0.05 was chosen as threshold.

### In situ hybridisation

Localization of *Ls*IR25a in copepodids, adults of both sexes and dissected male antennae was accomplished using in situ hybridization in the paraffin sections according to the previously published protocol [37] with some modifications. PCR products with T7 promoter on 5’ (sense) and 3’ (antisense) were used as templates for a single stranded digoxigenin (DIG)-labelled RNA probes synthesis (primers: IR25a-1_f and IR25a-1_r with and without T7 primer extension, S2 Table). Digoxigenin (DIG)-labelled sense (S) and antisense (AS) probes were prepared by in vitro transcription using the DIG RNA Labelling Kit (Roche). Hybridisations with sense probes were carried out as negative controls, while hybridisations with a known set of probes detecting LsTryp1 [37] served as a positive control. Probe concentration and quality was determined by spectrometry (Nanodrop ND-1000) and 1,5% agarose gel, respectively.

Briefly, paraffin sections of copepodids, adults and dissected male antennae were baked at 60°C for a minimum of 20 min and treated with Histoclear (National Diagnostics, Atlanta, GA, USA) prior to rehydration of tissue and proteinase K treatment for 20 min, followed by tissue fixation in 4% formaldehyde in PBS, acetic anhydride treatment and dehydration. Hybridization mix (100 ul) containing 50 ng of DIG-labelled RNA was added to the tissue and left overnight in a vacuum chamber at 70°C. DIG-labelled probes were visualised using secondary antibody labelled with an anti-DIG alkaline phosphatase-conjugated FAB fragment and a chromogen substrate containing nitroblue tetrazolium (NBT) (Roche Diagnostics GMbH, Mannheim, Germany) and 5-bromo-4- chloro-3-indolyl phosphate (BCIP) (Roche Diagnostics).

### Infection of host fish: Atlantic salmon (*Salmo salar*)

Atlantic salmon (*Salmo salar*), were placed in single fish tanks (70 liters) with constant sea water flow [38]. See water temperature was maintained at 10°C in course of whole experiment. During infection period (total of 10 minutes), water level was decreased to around 10 cm, to enhance contact between fish and copepodids. Fish were washed by 200 copepodids (7–9 days post hatching) and left without water flow for 10 minutes, with water manually mixed every 5 minutes. After that time, the water flow was restarted. From this moment (designated as 0h) detachment of copepodids was monitored closely. All copepodids not-attached during infection period and detached from fish were washed out from system with water flow, and collected into the nets. The nets were changed every 30 minutes for 4 hours from time 0h (beginning of water flow). Afterwards the nets were replaced every day up to 72h. Experiment was terminated two weeks after infection, and the attached lice were removed manually from anesthetized fish. To determine the infection efficiency, two-way ANOVA followed by the post hoc Tukey-Kramer test was applied.

### Infection of non-host fish: Lumpfish (*Cyclopterus lumpus*) and Ballan wrasse (*Labrus bergylta*)

Infection procedure of non-host fish was as described for Atlantic salmon with small modifications. Smaller tanks were used in order to enable the attachment process and to reliably evaluate attachment rate and retention time of copepodids on the fish.

Single fish from both tested species: lumpfish (*Cyclopterus lumpus*) and Ballan wrasse (*Labrus bergylta*), were placed in separate 2 liter boxes, with constant sea water flow. Sea water temperature was maintained at 10°C in course of the whole experiment. During infection period (10 minutes), water level was decreased to 5 cm to enhance contact between fish and copepodids. Fish were washed by 150 copepodids (7–9 days post hatching) and left without water flow, with water mixed every 5 minutes. After that time, all not-attached copepodids were carefully removed from the tank. The water flow was restarted 10 minutes after infection start. From this moment (designated as 0h) detachment of copepodids was monitored closely. All copepodids leaving the fish were collected into incubators with nets, both removed with water flow or manually at each time point, so they were not given a second chance to attach to fish. Incubators were changed every 30 minutes for 4 hours from time 0h (beginning of water flow). This period was sufficient for all control animals to leave system. Afterwards the incubators were changed every 24 hours. Experiment was terminated 72h post infection, and the attached lice were removed manually from anesthetized fish, to maintain their welfare. Fish were anesthetized using adapted salmon protocol [33]. To determine the infection efficiency, two-way ANOVA followed by the post hoc Tukey-Kramer test was used.

### Statistical analysis

Statistical evaluation of differences in mRNA level between the control group and the dsRNA treated groups, and between different stages/sexes, was performed by Independent-Samples T-Test. When more than two groups were compared to each other a two-way ANOVA followed by the post hoc Tukey-Kramer test was utilized. All statistical analyses were performed using SPSS Software (IBM SPSS Statistics for Macintosh, Version 23.0. Armonk, NY: IBM Corp.) and/or Excel 2011 (Microsoft Corp., Redmond, WA, USA). Exact information about which test was used for statistical analysis are indicated in the description of each experiment.

## Results

### Expression of IRs in various developmental stages

**Fig 1:**
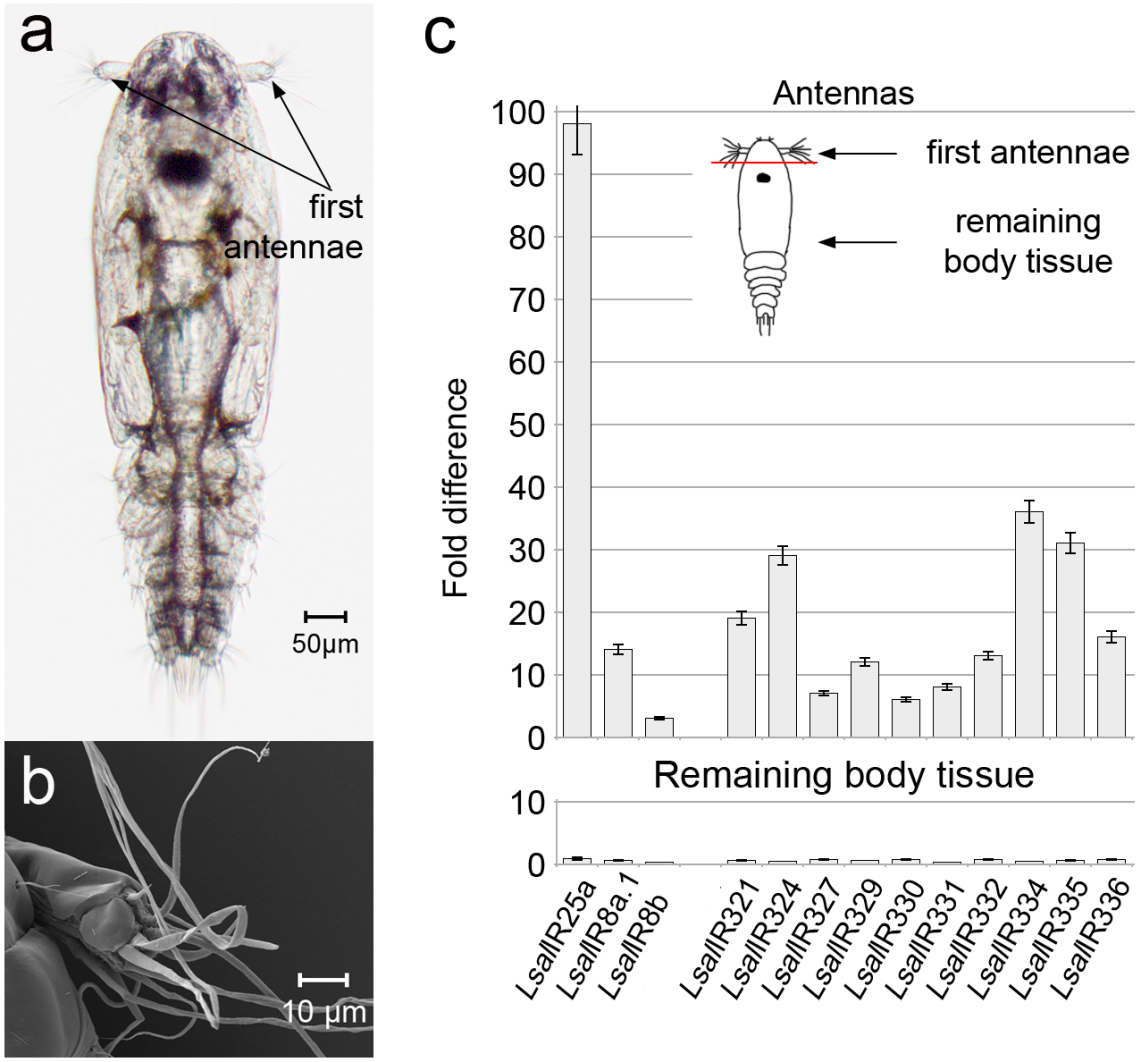
Copepodid, infectious stage of *L*.*salmonis* and antenna-specific expression of IRs: Co-receptors and antennal IRs. (**a**). Free living, infectious stage of *L*. *salmonis* with marked sensory organs—first antennae. (**b**). Close up of copepodid’s first antenna in SEM. Long sensilla are located mainly on the most distal segment. **(c)**. Expression of co-receptors and antennal IRs in antennae and remaining body tissue. Expression levels are given as a fold difference compared to intact animal. Bars indicate standard deviation. Analysis was performed on three parallels, where each antennal sample consisted of antennae pairs dissected from 500 animals, and the remaining body samples consisted of 100 dissected animals. On the right top, drawing of copepodid showing first antennae dissection for expression analysis.

**Fig 2:**
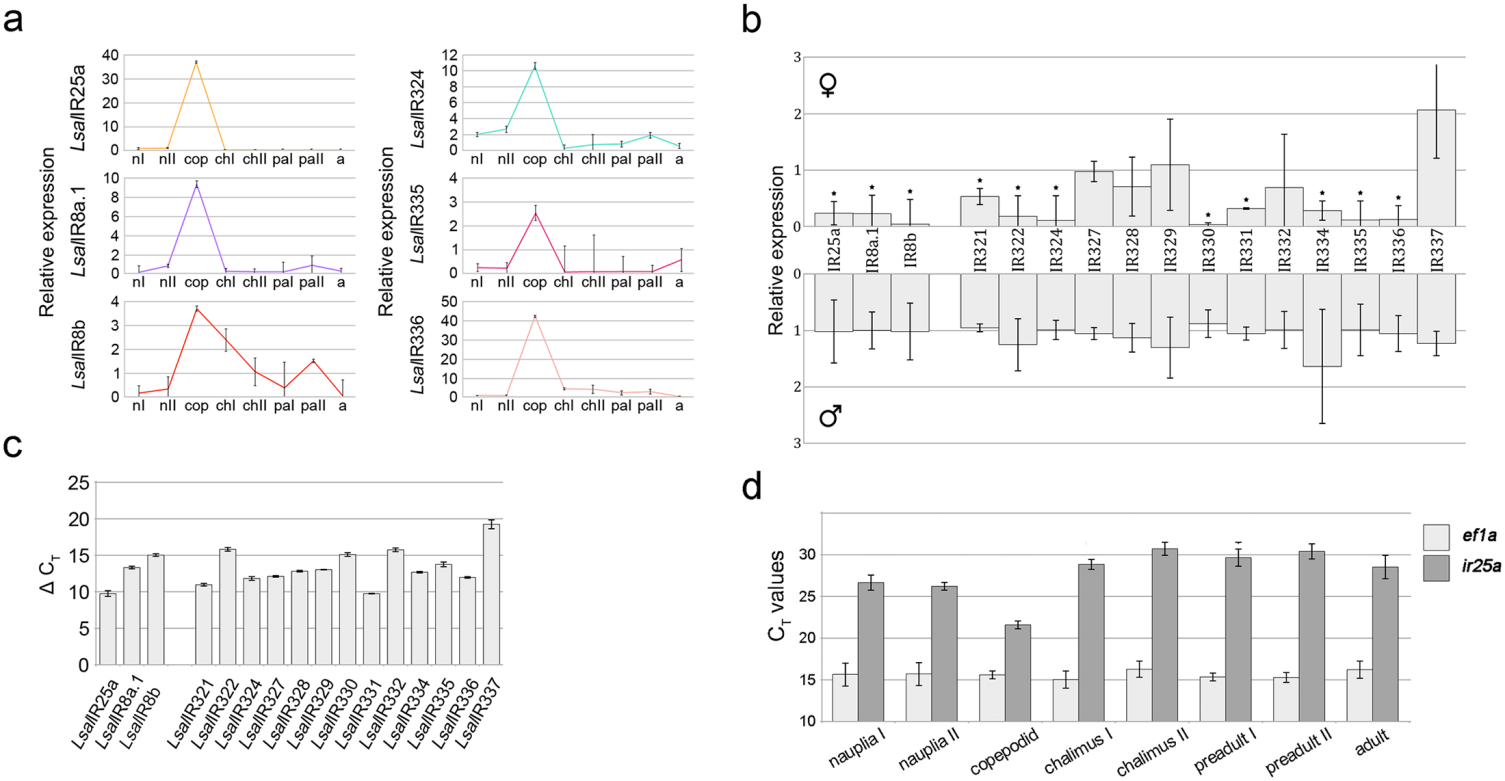
IRs expression levels through *L*. *salmonis* life cycle and in selected tissues in adult lice. (**a**). Relative expression level of three co-receptors and selected antennal IR genes in all developmental stages. The highest expression is in planktonic copepodids for all tested genes. Quantification was performed on batches of 100 nauplia I, nauplia II, copepodids, 20 chalimus I, 10 chalimus II, 4 preadult I females, 2 preadult II females, 1 adult female, 10 preadult I males, 4 preadult II males and 1 adult male (n = 5). Error bars show standard deviation. Abbreviations: nI—nauplia I, nII—nauplia II, cop—copepodid, chI—chalimus I, chII—chalimus II, paI—preadult I (both sexes), paII—preadult II (both sexes), a–adult (both sexes). (**b**). Comparison of transcripts level of antennal IRs in adults of both sexes tested by qRT-PCR. All co-receptors and 8 out of 13 antennal genes reveal higher expression in males than in females. Expression-PCR was performed on 1 adult female or male (n = 5). Error bars show standard deviation. Each louse was analysed separately and standard deviations represent individual differences. Asterisks indicate statistically significant differences in expression level between male and female (p < 0.05). Statistical analysis was performed using Independent-Samples T-Test. Comparison was performed for each gene independently. (**c**). Transcript level of IR genes in the copepodid stage. mRNA level for each tested gene is presented as a ΔC_T_ value. Expression-PCR was performed on batches of 100 copepodids (n = 5). Error bars show standard deviation. (**d**). Comparison of C_T_ values of *Ls*IR25a and reference gene *Ls*EF1A in all developmental stages. *Ls*EF1A is expressed at the same level in all developmental stages whereas the C_T_ values for *Ls*IR25a vary considerably and are the lowest in the copepodid stage. Q-PCR were done on same samples as in Fig 2a (n = 6).

Detailed identification and assessment of all IR-genes and the encoded proteins is presented elsewhere [31]. The focus of the present study is IRs expressed mainly in lice olfactory tissues (Fig 1a and 1b), three co-receptors and 13 potentially odor-specific IRs classified as antennal [31] (S1 Table). Full-length cDNA have been obtained for all genes by RACE and sequence analysis revealed that none of the antennal IRs has orthologues (S1 Table). Quantitative Real Time PCR (qRT-PCR) analysis of all the co-receptors and the antennal IRs (exception *Lsal*IR323 and *Lsal*IR325 due to lack of good quality sequence) confirmed that these genes are primarily expressed in the first antenna in the copepodids (Fig 1c). The expression dynamics for these genes were compared between various developmental stages (from nauplia I to adult male and female) by qRT-PCR. The differences were striking for all tested genes with significantly higher expression in the copepodids (Fig 2a). The expression levels of all co-receptors and tested antennal IRs are similar in preadult stages of both sexes. However, higher transcript levels for all co-receptors and eight antennal IRs (*Lsal*IR321, *Lsal*IR322, *Lsal*IR324, *Lsal*IR330, *Lsal*IR331, *Lsal*IR334, *Lsal*IR335 and *Lsal*IR336) were evident in the adult males (Fig 2b). However, the transcript levels for the IRs are far higher in copepodids than in the males (from 2.5 fold for *Lsal*IR335 to 127 fold for *Lsal*IR334). In general, the qRT-PCR analysis reveals low expression levels for all the tested IRs compared to the reference gene (*Lsal*EF1A) (Fig 2c).

### IRs expression in various tissues

**Fig 3:**
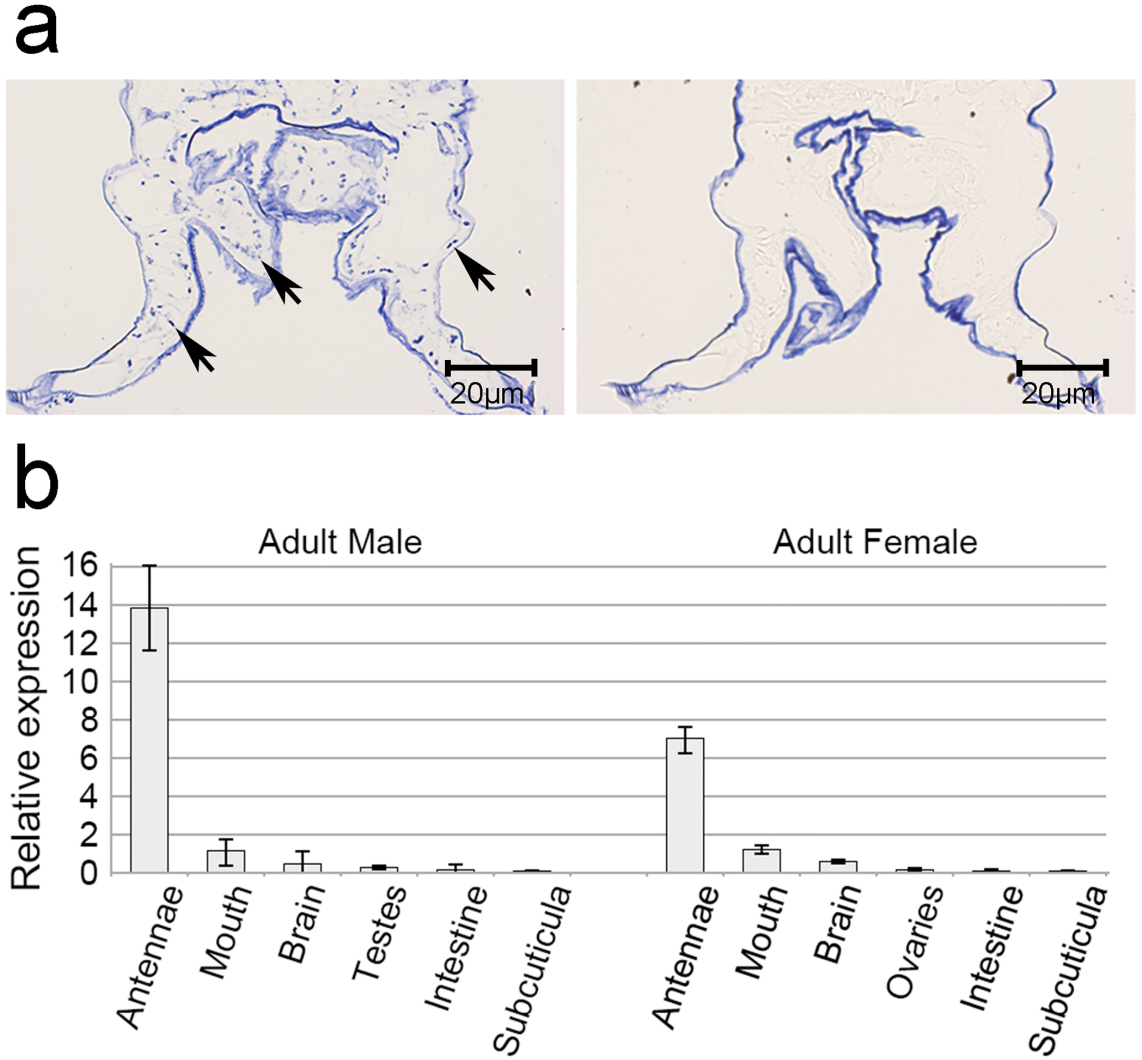
*Ls*IR25a expression in selected tissues from adult female and male. (**a**) In situ hybridization of paraffin sections of antennae dissected from adult salmon louse male. Antisense probes for *LsalIR25a* label neuronal cell bodies scattered in the male antennae. No specific labeling was detectable with the sense control. Specifically labeled cell bodies are indicated with an arrow. The cuticle in each section is non-specifically labeled in blue. (**b**) Q-PCR data shows relative expression in organs/tissues relative to the entire adult animal. *Lsal*IR25a is highest expressed in antennae. In the remaining body, the highest expression is detected in the mouth and the brain and low level of transcripts is detected in reproductive tissues (testes and ovaries), intestine and subcuticular tissue. Quantification of gene expression was performed on samples consisting of 10–20 dissected organs (n = 3). Error bars show standard deviation.

*Lsal*IR25a shows highest transcription of all *Lsal*IRs, therefore we have attempted to evaluate its expression pattern by in situ hybridization in paraffin sections of adults and copepodids. We were not able to detect *Lsal*IR25a transcripts in the whole body sections of tested stages, likely due to the small size of the animals (copepodids), restricted expression area of the gene, not included in the section or too low transcript level to be detectable by this method. RNAseq data indicate that the highest level of *Lsal*IR25a transcripts in adults is detected in the male antennae, which is very difficult to identify on the whole body section. To increase our chances of success we dissected antennae from numerous males, embedded in paraffin and used for in situ hybridization. With high concentration of DIG-labeled RNA probe (50ng/μl) we were able to detect single cell bodies scattered in the antennae (Fig 3a). Positive cells, likely olfactory receptor neurons (ONR), are located along the antennae, in close proximity of the surface.

To investigate expression pattern of *Lsal*IR25a in remaining tissues we have attempted to use immunohistochemistry with a polyclonal antibody on copepodids and adults. We were not able to obtain specific results by immunohistochemistry due to unspecific binding of the polyclonal antibody (verified by Western Blot).

Therefore, we dissected various organs/tissues from adults of both sexes and examined it by Q-PCR. *Lsal*IR25a transcripts were detected in all tested tissues at various levels. The highest expression of *Lsal*IR25a was found in the antennae (Fig 3B), what is in an agreement with in situ results and RNAseq data (unpublished data). Based on these results, we conclude that *Lsal*IR25a is likely to function as a main co-receptor, and might be involved in fulfilling diverse functions of the IR system in *L*. *salmonis*.

### RNAi knock-down experiment on nauplia I

**Fig 4:**
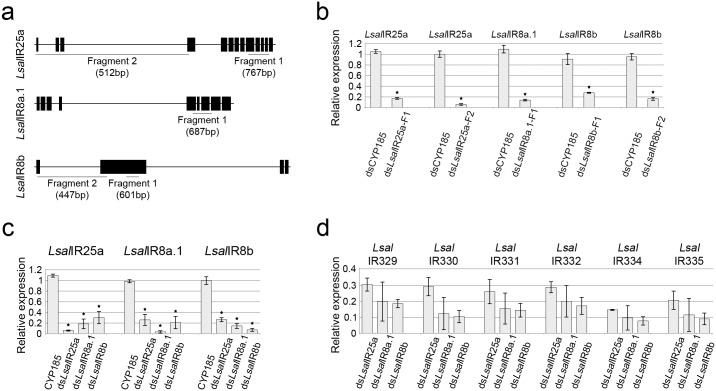
Treatment with dsRNA against co-receptors. (**a**) Exon/intron organization of IR co-receptor with underlined regions targeted by dsRNA. Fragments are complementary to exon sequences (black bars) only. Size of each fragments are given in brackets. For *Lsal*IR25a and *Lsal*IR8b two different fragments were used. (**b**) Silencing efficacy for each tested fragment (F) is presented as a relative expression of targeted gene. Type of treatment is indicated under each bar, and the gene tested above each graph. Error bars shows standard deviation. Each batch contained 100 copepodids, n = 6 and the experiment was repeated 3 times for each gene. Asterisks show significant differences (p < 0,05). Statistical evaluation of differences in mRNA level between the control group and the dsRNA treated group, was performed by Independent-Samples T-Test independently for each gene. (**c**) Mutual interactions between co-receptors. Expression levels (given as relative expression) for all co-receptors were tested for each dsRNA treatment. The type of dsRNA treatment is given under each bar and tested genes are given above each graph. Error bars indicate standard deviation. Each batch contained 100 copepodids, n = 5. Each experiment was repeated 3 times. Asterisks indicate significant difference (p < 0.05). Statistical evaluation of differences in mRNA level of each gene between the control group and the dsRNA treated group, was performed by Independent-Samples T-Test, for each gene independently. (**d**) The influence of co-receptors down-regulation on antennal IRs. Six genes show the lowest expression levels in samples treated with ds*Lsal*IR8b. Tested genes indicated above graph, type of treatment stated under each bar. Graphs show relative expression of each gene in comparison to the control, treated with dsCYP185. Expression of each gene in the control sample was set as 1 and omitted in this graph for clarity. Error bars show standard deviation. Each batch contained 100 copepodids, n = 4. Experiment was repeated 4 times.

To elucidate the function of IRs in chemoreception in *L*. *salmonis* we have applied RNA interference (RNAi) using double-stranded RNA (dsRNA) [34]. A previous study has demonstrated that there is a reciprocal requirement of pairs of co-receptors and antennal odor-specific IRs for proper receptor targeting to sensory cilia and signal transduction [39]. We have down-regulated all three co-receptors to investigate their influence on each other and on antennal IRs (see Fig 4). To diminish possibility of cross-reactivity of dsRNA we selected fragments with the lowest possible similarity to the other salmon louse IRs (S1 Fig). All dsRNA fragments (Fig 4a) gave highly significant (p < 0,05) down regulation of the target genes giving between 80% (for *Lsal*IR25a Fragment-1) and nearly 100% (for *Lsal*IR8a.1) reduction in mRNA levels (Fig 4b). For *Lsal*IR25a and *Lsal*IR8b both fragments for each gene gave gene silencing but the strongest down-regulation was seen for the fragments located at the 5’ end of the genes (*Lsal*IR25a Fragment-2 and *Lsal*IR8b Fragment-2) (Fig 4b).

Down regulation of a given co-receptor influence the expression level of other co-receptors. RNAi against *Lsal*IR25a (Fragment-2), *Lsal*IR8a.1 and *Lsal*IR8b (Fragment-2) resulted in significant (p < 0,05) silencing of the remaining co-receptors (Fig 4c) whereas no such down-regulation was evident for forth co-receptor present in *L*.*salmonis Lsal*IR8a.2 (data not shown). These results indicate that *Lsal*IR25a, *Lsal*IR8a.1 and *Lsal*IR8b have a significant function in regulating transcripts levels of other IR co-receptor genes and their expression is mutually dependent. Although down-regulation of co-receptors influence expression levels of other co-receptors (e.g. by causing down-regulation), significantly stronger effect for each gene is observed when specific dsRNA is used (Fig 4c).

Effects of co-receptors down-regulation by RNAi on the antennal IRs were assessed by qRT-PCR and showed that down-regulation of *Lsal*IR25a, *Lsal*IR8a.1 and *Lsal*IR8b significantly affects expression of the antennal odor-specific IRs. All antennal IRs were significantly down-regulated (p < 0,05) when co-receptor transcripts were eliminated (S2 Fig). The strongest effects for the six tested genes were observed when copepodids were treated with ds*Lsal*IR8b (Fig 4d).

Based on the mutual interactions between co-receptors and antennal receptors, we propose that *Lsal*IR25a, *Lsal*IR8a.1 and *Lsal*IR8b are the main players in regulating proper IR expression and, hence the function for the IRs. Although each of these co-receptors regulates expression of all tested antennal IRs, the lowest levels of transcripts were observed in animals treated with ds*Lsal*IR8b (Fig 4f).

### Infection of salmon (*Salmo salar*) after RNAi

In order to explore the impact of IRs in salmon louse, we infected Atlantic salmon (*Salmo salar*) with copepodids after treatment with dsRNA against co-receptors having significant effect on the antennal receptors: *Lsal*IR25a, *Lsal*IR8a.1 and *Lsal*IR8b (see Fig 4d). After infection not-attached copepodids were collected during a 72h period and the final number of attached lice was assessed two weeks post infection.

**Fig 5:**
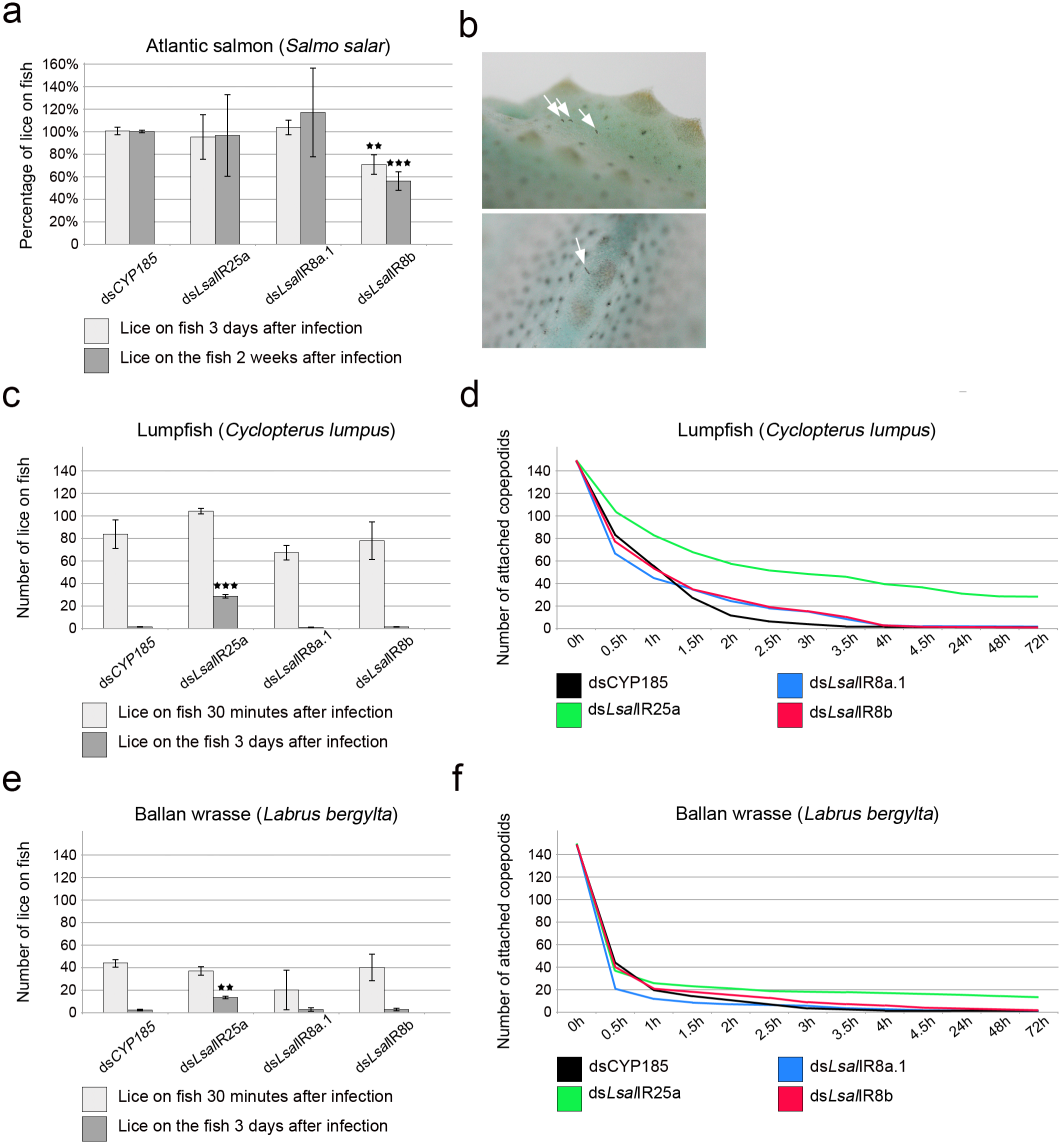
Co-receptor RNAi and infection efficiency of various fish species. (**a**). Infection of Atlantic salmon (*Salmo salar*) given as percentage copepodids attached at three days and two weeks post infection. Significant reduced attachment (day three post infection) and settlement (two weeks post infection) was observed for the group treated with ds*Lsal*IR8b. Type of dsRNA treatment given under each bar. (**b**) Copepodids attached to the lumpfish 72h post infection (whit arrows). (**c**) Infection on the non-host Lumpfish (*Cyclopterus lumpus*). Number of copepodids attached to lumpfish 30 minutes and three days post infection. The group treated with ds*Lsal*IR25a showed significantly higher infection three days post infection. Type of dsRNA treatment displayed under each bar. (**d**) Number of copepodids attached to the Lumpfish during the experiments (from infection to 72h post infection). Average values for each time point are used. Each line indicates different types of dsRNA used (color code under the graph). (**e**) Number of copepodids attached to Ballan wrasse (*Labrus bergylta*) at 30 minutes post infection and three days post infection. Type of dsRNA used displayed under each bar. (**f**) Number of copepodids attached to the Ballan wrasse during the course of the experiments (from infection to 72h post infection). Average values for each time point are shown. Each line indicates different types of dsRNA used (color code under the graph). Each experiment was repeated between 3 and 6 times (n = 3–6) using copepodids treated with dsRNA (200 for Atlantic salmon, 150 for lumpfish and Ballan wrass). Asterisks show various levels of significance between the control group and each RNAi treated groups evaluated with two-way ANOVA followed by the post hoc Tukey-Kramer test: 2 asterisks (p < 0.01), 3 asterisks (p < 0.0001).

Attachment efficiency evaluated 3 days post infection revealed no or very small variation between groups of animals treated with control dsRNA, *Lsal*IR8a.1 or *Ls*IalR25a (Fig 5a). The attachment success of copepodids treated with *Lsal*IR8b dsRNA was significantly reduced giving 30% fewer copepodids (p < 0.01) (Fig 5a). The average number of lice recovered from fish two weeks post infection was highest in the group treated with dsRNA against *Ls*IR8a.1. The number of lice recovered in the *Lsal*IR8b dsRNA group was significantly reduced (p < 0.0001) (Fig 5a) indicating that L*sal*IR8b is important to recognize host fish before attachment and settlement.

### Infection of non-host fish

To demonstrate the significance of IR co-receptors in shaping salmon louse host specificity the lumpfish (*Cyclopterus lumpus*) were infected with copepodids incubated with dsRNAs against co-receptors. Copepodids were given 30 minutes to find a host and attach. After that time point not-attached copepodids were carefully removed from the system and counted, leaving only copepodids attached to the fish in the system. This point was set as 0h and detachment of copepodids was closely monitored and all freely swimming lice were collected at every time point during the next 72 hours.

Copepodids from all groups attached to the lumpfish but at different levels (9–69%, see Fig 5c). The highest affinity was for the group treated with dsRNA against *Lsal*IR25a with an average of 104 copepodids/fish (69% attachment) (Fig 5c and 5d). Control copepodids stayed on the fish for a very short time and more than 91% of the attached copepodids were leaving the fish within the first two hours after infection and 98% within 4 hours post challenge (Fig 5d). The same pattern was observed for the groups treated with *Lsal*IR8a.1 dsRNA and *Lsal*IR8b dsRNA where copepodids left the fish within 4 hours post infection (over 98% copepodids in both groups) (Fig 5d). A significant number of copepodids treated with *Lsal*IR25a dsRNA stayed on the lumpfish for a longer time and 50% of the attached copepodids were present on the fish 2 hours after infection, and 36% 4 hours post infection (Fig 5c and 5d). A significant number of copepodids from this group were observed on lumpfish both at 24 hours (33.4%) and at 72 hours post infection (27.5%) (Fig 5c and 5d). No significant difference in early attachment (i.e. 30 minutes) was seen between the different RNAi groups (Fig 5c) but a significant proportion (p < 0.0001) (Fig 5b and 5c) of the *Lsal*IR25a RNAi copepodids were recovered on the lumpfish 72h post infection.

To further validate the results from the lumpfish experiment where down regulation of *Lsal*IR25a co-receptor affects salmon louse copepodids infection pattern we repeated the experiment using Ballan wrasse (*Labrus bergylta*) another non-host fish species. Copepodids from all groups attached to Ballan wrasse at a lower level compared to the lumpfish (average 20–42%) but the overall pattern was very similar (Fig 5e and 5f). No increase in attachment frequency was observed for the *Lsal*IR25a dsRNA treated group 30 minutes post infection (Fig 5e). However, *Lsal*IR25a dsRNA treated animals stay on the fish longer compared to the other groups and after 2 hours 13% of *Lsal*IR25a dsRNA treated animals were still on the fish compared to 5.5% from the control group, 4% from *Lsal*IR8a.1 dsRNA and 8.7% from *Lsal*IR8b dsRNA treated group. After 4 hours 11% of the copepodids from *Lsal*IR25a dsRNA treated group were still attached to the fish, in contrast to 1.5% for the control and 3% for both *Lsal*IR8a.1 and *Lsal*IR8b dsRNA groups (Fig 5f). Similar to the lumpfish experiment the copepodids treated with *Lsal*IR25a dsRNA stay significantly longer (p < 0.001, Fig 5e) on the Ballan wrasse compared the other groups and at 72 hour post infection 9% of copepodids were still attached (1.5% in control, 2% in *Ls*IR8a.1 and 2.5% in *Ls*IR8b groups) (Fig 5e and 5f). This indicates that the co-receptor *Lsal*IR25a has important function in discrimination between host and non-host fish.

## Discussion

During a previous study [31] 39 genes belonging to Ionoropic Glutamate Receptors (iGluRs) were identified and of these 27 belong to Ionotropic Receptors (IRs). In the absence of the classical seven transmembrane receptors (GRs and ORs), typically found in other Arthropods, IRs seems to be the only candidates involved in chemical detection. Based on the protein sequence and phylogenetic analysis, *Lsal*IRs were categorized into two groups consisting of the highly conserved co-receptors (*Lsal*IR25a and three paralogues of *Lsal*IR8a) and the odor-binding IRs [31]. For only a few *Lsal*IRs (*Lsal*IR25a, *Lsal*IR8a.1, *Lsal*IR8a.2, *Lsal*IR8b, *Lsal*IR93a and *Lsal*IR21a) orthologes were identified whereas the odor-binding *Lsal*IRs are clearly distinct from IRs in other species [12,16,31,32,40–42]. The lack of obvious orthologues between the odor-binding *Lsal*IRs and those in other species suggests specialization of *L*. *salmonis* in detecting a defined range of chemical cues. This could be a consequence of the adaptation to a parasitic life style and a high degree of host specificity. As a parasite on salmonids the salmon louse must be able to distinguish between suitable and non-suitable hosts. The gene expression data for the included IRs shows a striking pattern where the highest expression takes place in the infectious copepodids and localized to the antennae (Figs 1c and 2a). Although at lower level, the same pattern occurs in adult lice where there is high expression in antennae compared to the other examined tissues (see Fig 3) with a significantly higher expression in males (see Fig 2b). The same pattern is evident for both co-receptors and the putative odor-specific antennal IRs. The striking antennae associated expression pattern suggest that these IRs are involved in sensing environmental derived molecules (e.g. present on the host) in a similar way as other species [6,43]. For the copepodids this could be a part of the host recognition system.

Previously, it has been demonstrated that the expression and localization of co-receptors and odor-specific antennal IRs have reciprocal requirements [39], and that the odor-specific IRs works together with the co-receptors in order to mediate chemical signals [5,44]. Lack of the co-receptors affects proper localization of the odor-specific IRs in the sensory cilia [39]. Through the RNAi experiments, we show that down-regulation of the co-receptors efficiently affects expression of the odor-specific IRs in the antennae (Fig 4d). However we were not able to evaluate changes in their localization by *in situ* hybridization or immunohistochemistry due to technical issues. The intensity of the antennal IRs down-regulation seems to be proportional to the level of *Lsal*IR8b (See Fig 4c and 4d). Moreover, we suggest that down-regulation of the antennal IRs observed in animals treated with ds*Lsal*IR25a and ds*Lsal*IR8a.1 could be a secondary effect of reduced *Lsal*IR8b levels.

The RNAi experiments indicate that there is a mutual dependence between the co-receptors. We measured a strong dependence between *Lsal*IR25a, *Lsal*IR8a.1 and *Lsal*IR8b, whereas the forth co-receptor present in *L*.*salmonis*, *Lsal*IR8a.2 [31], has low or no visible influence on the other co-receptors. Interestingly, *Lsal*IR8a.2 is absent in the antennae in any of the stages, but is expressed predominantly in adult stage of both sexes [31].

The ability to recognize the right host is crucial for parasites, especially species with a narrow host range like salmon louse. It is known that arthropods (e.g. mosquito [45,46], stable fly [43], fruit fly and endoparasitoid wasps [17]) utilize Olfactory and/or Gustatory Receptors to localize potential food or places for oviposition. In order to investigate the significance of IRs in host recognition we have used an assay involving live fish and not artificial approaches like the Y-tube assay applied in some previous studies [26,28]. While Y-tube assay allow evaluation of attractants/repellents, it does not enable to evaluate short-range recognition and subsequent discrimination processes.

In the present study, we demonstrated that knocking down IRs alters settlement of *L*. *salmonis* copepodids, both on the preferred host and non-hosts. Silencing of *Lsal*IR8b alter normal attachment and settlement rate of salmon lice on Atlantic salmon (the normal host), whereas this has no effect on discrimination between host and non-host fish. This suggests that deficiency of *Lsal*IR8b negatively affects the lice’s ability to recognize Atlantic salmon (see Fig 5a). Knock down of *Lsal*IR25a has no negative effect on identification of the Atlantic salmon since copepodids’ attachment and settlement was comparable to control animals (see Fig 5a). However, ds*Lsal*IR25a treated lice showed significant difficulties in discriminating between host and non-host fish (see Fig 5c–5f).

The ability to attach to non-salmonid hosts by salmon louse has been shown before [47,48]. However, when the copepodids are given a choice of host and a non-host fish (Atlantic salmon and cod or saith), significantly more copepodids settle on Atlantic salmon. In addition, the migration from non-host fish to salmon continues for up to 4 hours post-infection, suggesting that discrimination between host/non-host takes place for a while after settlement [48]. In our experiments, this time was also sufficient for control, ds*Lsal*IR8a.1 and ds*Lsal*IR8b lice to recognize non-host fish and they left within four hours (Fig 5d and 5f). However, a significant proportion of the ds*Lsal*IR25a treated copepodids stayed on wrong host at least for 72 hours (Fig 5c–5f) indicating difficulties in recognizing that they were on wrong host. This result suggests that *Lsal*IR25a has important function in the ability to distinguish between different fish species by mediating chemical cues connected with the host.

The outcome of our experiments indicates that the lack of *Lsal*IR25a changes host’s preferences, enabling infection on non-host fish, likely making lice less targeted and specific to salmon smell. Therefore, *Lsal*IR25a seems to be involved in mediating a primary signal host/non-host, which needs further validation by the lice.

On the other hand, knock down of *Lsal*IR8b resulted in a significant reduction in the settlement on Atlantic salmon. This suggests that *Lsal*IR8b either directly or indirectly via odor-specific IR, is involved in secondary decision-making to recognize salmon-specific features. We hypothesize that lice lacking *Lsal*IR8b are unable to confirm that they are attached to the right host, and consequently they leave any fish, including Atlantic salmon. Although lower levels of *Lsal*IR8b are observed in animals treated with dsRNA against other co-receptors, we do not observe similar alterations in host preference. Therefore, we suggest that the level of available transcripts of *Lsal*IR8b is an important factor in formation of behavioral response. It is likely that the effect is visible only if transcript levels drop below a certain threshold, where protein production is insufficient to supply olfactory neurons with a proper signal. In such situations, it might be difficult/impossible to form functional pairs of co-receptor *Lsal*IR8b-odor-specific IR to efficiently transduce right signal about the host.

We conclude that both *Lsal*IR25a and *Lsal*IR8b have important function in the host/non-host discrimination process, where *Lsal*IR25a serves as a primary decision maker and *Lsal*IR8b verifies host recognition. The co-receptors work together with the odor-specific IRs in order to signal environmental cues. Chemical cues bound by the odor-specific IRs for host recognition in *L*.*salmonis* remain unknown but the present study clearly point towards the significance of IRs. Our findings open for the possibility to utilize the underlying host-specificity to develop new tools for salmon louse control.

## Supporting information

S1 Fig Multiple alignments between three co-receptors used in this study. dsRNA used are complementary to the areas marked in color. (**a**) 5’ end of co-receptors, complementary to fragments *Ls*IR25a-F2 (purple), *Ls*IR8b-F2 (grey). (**b**) Co-receptors sequence complementary to fragments *Ls*IR25a-F1 (yellow), *Ls*IR8b-F1 (green), *Ls*IR8a.1-F1 (blue). Identical base pairs are marked with asterisks under alignment.
(TIF)

S2 Fig Interactions between co-receptors and all tested here antennal odor-specific receptors. Down-regulation of co-receptors affects expression of all antennal IRs to various degrees. Type of dsRNA treatment is indicated above each graph and tested gene under each bar. Graphs show relative expression of each gene in comparison to the control, treated with dsCYP185. Expression of each gene in the control sample was set as 1 and omitted in this graph for clarity. Error bars indicate standard deviation. Each batch contained 100 copepodids, n = 5. Experiment was repeated 3 times. Asterisks indicate significant difference between the control and the test sample: p < 0,05. Statistical evaluation of differences in mRNA level between the control group and the dsRNA treated group, was performed for each gene separately by Independent-Samples T-Test, for each gene independently.
(TIF)

S1 Table List of tested *L*.*salmonis* IRs. These IRs have the highest expression level in the copepodid stage and are classified as antennae type expression profile. Stable IDs (EMLSAG) are given if available. Division between co-receptors and antennal genes is based on the structural differences and functional data. In blue are the genes where only partial sequence has been obtained.
(DOCX)

S2 Table List of primers used to produce templates for dsRNA and in situ hybridization. Templates were amplified with primers listed in the table. Second pair of primers was used to add T7 RNA polymerase binding sites on each end of the template (See Materials and methods).
(DOCX)

S3 Table List of primers used in qRT-PCR. All primer sets are located outside of the sequence complementary to the dsRNA used in the RNAi experiments.
(DOCX)
