# Supplementary figures and images for: Ionotropic receptors signal host recognition in the salmon louse (Lepeophtheirus salmonis, Copepoda)
Source: PLoS One. 2017 Jun 5;12(6):e0178812. doi: 10.1371/journal.pone.0178812 (PMC5459451; doi:10.1371/journal.pone.0178812)

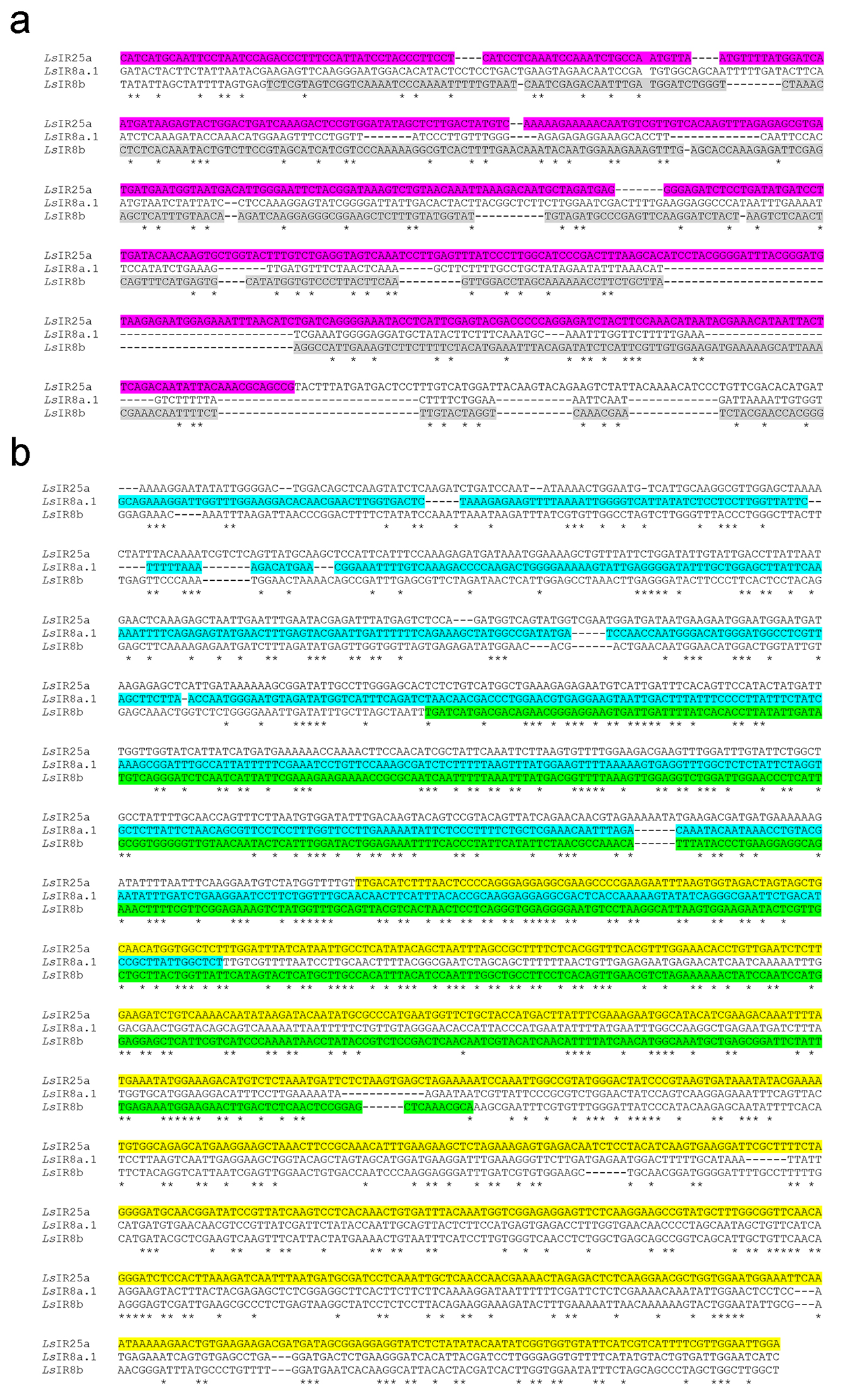

Supplement: S1 Fig — dsRNA used are complementary to the areas marked in color. (a) 5’ end of co-receptors, complementary to fragments LsIR25a-F2 (purple), LsIR8b-F2 (grey). (b) Co-receptors sequence complementary to fragments LsIR25a-F1 (yellow), LsIR8b-F1 (green), LsIR8a.1-F1 (blue). Identical base pairs are marked with asterisks under alignment. (TIF) [file pone.0178812.s001.tif]

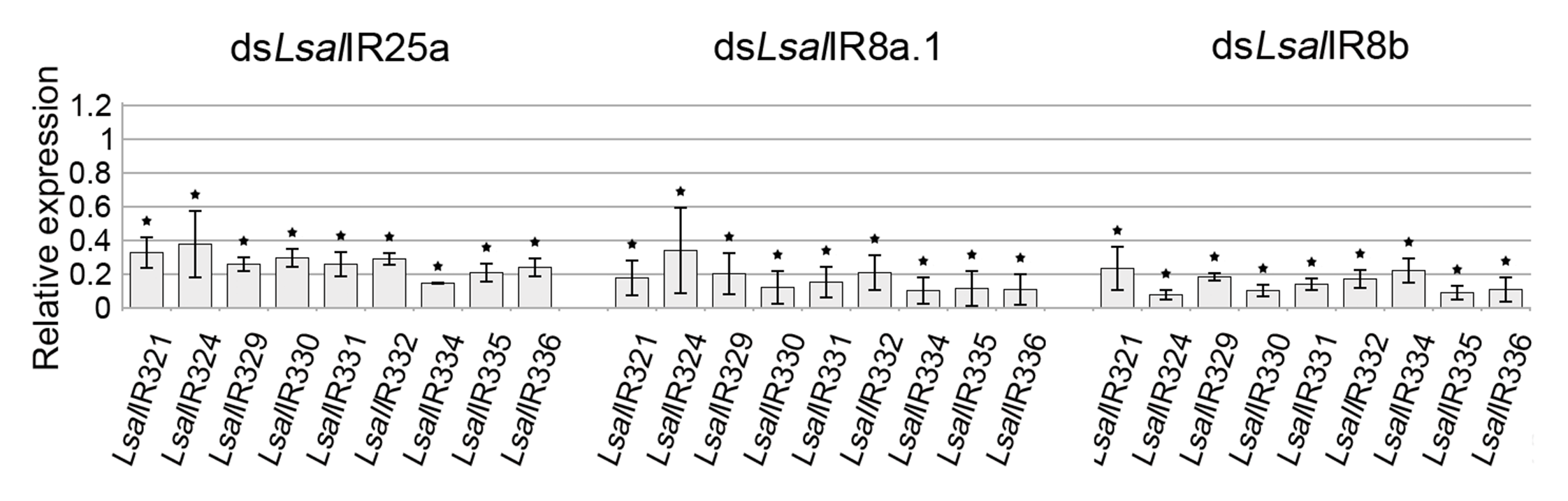

Supplement: S2 Fig — Down-regulation of co-receptors affects expression of all antennal IRs to various degrees. Type of dsRNA treatment is indicated above each graph and tested gene under each bar. Graphs show relative expression of each gene in comparison to the control, treated with dsCYP185. Expression of each gene in the control sample was set as 1 and omitted in this graph for clarity. Error bars indicate standard deviation. Each batch contained 100 copepodids, n = 5. Experiment was repeated 3 times. Asterisks indicate significant difference between the control and the test sample: p < 0,05. Statistical evaluation of differences in mRNA level between the control group and the dsRNA treated group, was performed for each gene separately by Independent-Samples T-Test, for each gene independently. (TIF) [file pone.0178812.s002.tif]
