# Supplementary material for: Ionotropic receptors signal host recognition in the salmon louse (Lepeophtheirus salmonis, Copepoda)
Source: PLoS One. 2017 Jun 5;12(6):e0178812. doi: 10.1371/journal.pone.0178812 (PMC5459451; doi:10.1371/journal.pone.0178812)
Supplement: S1 Table — These IRs have the highest expression level in the copepodid stage and are classified as antennae type expression profile. Stable IDs (EMLSAG) are given if available. Division between co-receptors and antennal genes is based on the structural differences and functional data. In blue are the genes where only partial sequence has been obtained. (DOCX) [file pone.0178812.s003.docx]

| **ASSIGNED NAME** | **STABLE ID** | **TYPE** | **FUNCTION** |
| --- | --- | --- | --- |
| *Lsal*IR25a | EMLSAG00000004146 | A | co-receptor |
| *Lsal*IR8b | EMLSAG00000003971 | A | co-receptor |
| *Lsal*IR8a.1 | EMLSAG00000002010 | A | co-receptor |
| *Lsal*IR321 | EMLSAG00000010025 | A | antennal |
| *Lsal*IR322 | g3871_sanger-strict.final.scaffolds | A | antennal |
| *Lsal*IR323 | EMLSAG00000012055 | A | antennal |
| *Lsal*IR324 | EMLSAG00000000121 | A | antennal |
| *Lsal*IR325 | g10896_sanger-strict.final.scaffolds | A | antennal |
| *Lsal*IR327 | g11484_sanger-strict.final.scaffolds | A | antennal |
| *Lsal*IR328 | g12240_sanger-strict.final.scaffolds | A | antennal |
| *Lsal*IR329 | g12444_sanger-strict.final.scaffolds | A | antennal |
| *Lsal*IR330 | g18627_sanger-strict.final.scaffolds | A | antennal |
| *Lsal*IR331 | g18792_sanger-strict.final.scaffolds | A | antennal |
| *Lsal*IR332 | g21611_sanger-strict.final.scaffolds | A | antennal |
| *Lsal*IR334 | EMLSAG00000004382 | A | antennal |
| *Lsal*IR335 | EMLSAG00000011162 | A | antennal |
| *Lsal*IR336 | EMLSAG00000011840 | A | antennal |
| *Lsal*IR337 | EMLSAG00000012109 | A | antennal |
