# Supplementary material for: Ionotropic receptors signal host recognition in the salmon louse (Lepeophtheirus salmonis, Copepoda)
Source: PLoS One. 2017 Jun 5;12(6):e0178812. doi: 10.1371/journal.pone.0178812 (PMC5459451; doi:10.1371/journal.pone.0178812)
Supplement: S2 Table — Templates were amplified with primers listed in the table. Second pair of primers was used to add T7 RNA polymerase binding sites on each end of the template (See Materials and methods). (DOCX) [file pone.0178812.s004.docx]

| **ASSIGNED NAME** | **STABLE ID** | **FRAGMENT** | **PRIMER NAME** | **PRIMER SEQUENCE** | **PRODUCT SIZE** |
| --- | --- | --- | --- | --- | --- |
| *Lsal*IR25a | EMLSAG00000004146 | Fragment 1 | IR25a-1_f | TTGACATCTTTAACTCCCCAGGGAGG | 767bp |
|  |  |  | IR25a-1_r | CCAATTCCAACGAAAATGACGATGA |  |
|  |  | Fragment 2 | IR25a-2_f | CATCATGCAATTCCTAATCCAGACCC | 512bp |
|  |  |  | IR25a-2_r | CGGCTGCGTTTGTAATATTGTCTGAA |  |
| *Lsal*IR8b | EMLSAG00000003971 | Fragment 1 | IR8b-1_f | TGATCATGACGACAGAACGGGAGGAA | 601bp |
|  |  |  | IR8b-1_r | TGCGTTTGAGCTCCGGAGTTGAGA |  |
|  |  | Fragment 2 | IR8b-2_f | TCTCGTAGTCGGTCAAAATCCCA | 447bp |
|  |  |  | IR8b-2_r | CCCGTGGTTCGTAGATTCGTTTGA |  |
| *Lsal*IR8a.1 | EMLSAG00000002010 | Fragment 1 | IR8a.1-1_f | GCAGAAAGGATTGGTTTGGA | 687bp |
|  |  |  | IR8a.1-1_r | AGCCAATAAGCGGATGTCAG |  |
