# Supplementary material for: Ionotropic receptors signal host recognition in the salmon louse (Lepeophtheirus salmonis, Copepoda)
Source: PLoS One. 2017 Jun 5;12(6):e0178812. doi: 10.1371/journal.pone.0178812 (PMC5459451; doi:10.1371/journal.pone.0178812)
Supplement: S3 Table — All primer sets are located outside of the sequence complementary to the dsRNA used in the RNAi experiments. (DOCX) [file pone.0178812.s005.docx]

| **ASSIGNED NAME** | **STABLE ID** | **PRIMER NAME** | **PRIMER SEQUENCE** | **PRODUCT SIZE** |
| --- | --- | --- | --- | --- |
| *Lsal*IR25a | EMLSAG00000004146 | b1565 | AGAGTTTCAGGACGGTGCTCCAA | 209bp |
|  |  | b1566 | GAGCTGTCCAGTCCCCAATATATTCC |  |
| *Lsal*IR8b | EMLSAG00000003971 | b1899 | GGGAAAATCCGAGGGCTCAAGTATA | 170bp |
|  |  | b1900 | AACCCAAGACTAGGCCAACACGATA |  |
| *Lsal*IR8a.1 | EMLSAG00000002010 | b1549 | CCTCCTTCAAAAGTCGATTCCAAGAA | 198bp |
|  |  | b1241 | TAGAACAATCCGATGTGGCAGCAA |  |
| *Lsal*IR321 | EMLSAG00000010025 | b2907 | TCGTGATTCAGCAGATCAATCCAA | 221bp |
|  |  | b2908 | TGTAATAGGGAATGAGCATTCGGC |  |
| *Lsal*IR322 | g3871_sanger-strict.final.scaffolds | b2889 | AGACGGGTCCCGCAGGAATAT | 129bp |
|  |  | b2890 | TTCCCAGAATCCCCGACTCATT |  |
| *Lsal*IR324 | EMLSAG00000000121 | b1541 | TTTGGAGAGGTATGAAGTGGTCGCTT | 192bp |
|  |  | b1542 | TGGCGAAACGCGTAGAGATTAACA |  |
| *Lsal*IR327 | g11484_sanger-strict.final.scaffolds | b2887 | ATATTTCGCAACCCATGACGTCAA | 186bp |
|  |  | b2888 | TGGGTAAAGGGGATTCTGCAACTT |  |
| *Lsal*IR328 | g12240_sanger-strict.final.scaffolds | b2895 | AATGGAATGGTCGGAATGGTTCAA | 184bp |
|  |  | b2896 | TTCCAAAAGGATTCAACATGGCTC |  |
| *Lsal*IR329 | g12444_sanger-strict.final.scaffolds | b2358 | CGACACTCACTCCTGGTACGTGATCATT | 193bp |
|  |  | b2359 | CCACATAGTTAAAAGGATCCGTTCCGAA |  |
| *Lsal*IR330 | g18627_sanger-strict.final.scaffolds | b2891 | CTTTTGCTCACCCCTCATTGCATT | 131bp |
|  |  | b2892 | CCACGGCATCAAGACAGGATACAA |  |
| *Lsal*IR331 | g18792_sanger-strict.final.scaffolds | b2885 | TTGAGAGATAATGCACCTCGAACCTT | 171bp |
|  |  | b2886 | TTCAAGCCGATGCTTCAGGAATC |  |
| *Lsal*IR332 | g21611_sanger-strict.final.scaffolds | b1545 | CGTCCCCAAATGTGGATTCCAA | 160bp |
|  |  | b1546 | CACCTCTGGCTTGATCATCTTCGTC |  |
| *Lsal*IR334 | EMLSAG00000004382 | b2901 | GAGGCGGGGGAATGTCATGTT | 128bp |
|  |  | b2902 | TCCTTAAGGCTTGGGCTCATCC |  |
| *Lsal*IR335 | EMLSAG00000011162 | b1556 | CCGATTTGCCAATAGACGTAGAGGAT | 118bp |
|  |  | b1557 | TGGCGATTTTTCTTCCATGTGGT |  |
| *Lsal*IR336 | EMLSAG00000011840 | b1561 | GAGTATCAACTCATTCTCCTCAACGGAA | 133bp |
|  |  | b1562 | TTTCTTCTTGTCCTTCGATGAGCTTG |  |
| *Lsal*IR337 | EMLSAG00000012109 | b2897 | CCCATGCAAATGGTACAAGTGGAT | 130bp |
|  |  | b2898 | TGATTTGCCCCAATGCGTCTAA |  |
